# Alectinib-induced rash unresponsive to desensitization: a case report and literature review

**DOI:** 10.1186/s12890-023-02558-6

**Published:** 2023-07-15

**Authors:** Robin Raquel Rodriguez, Jessie Dindak, Janet Kline, Moses S. Raj

**Affiliations:** Department of Medical Oncology, Allegheny Health Network, Pittsburgh, PA USA

**Keywords:** Targeted therapy, ALK gene mutation, Non-small cell lung cancer, Rash

## Abstract

**Background:**

Since the inception of targeted therapies in treating lung cancer, providers have had to be aware of a new host of side effects when selecting management options for patients. Although targeted therapies are creating increased hope for patients with non-small cell lung cancers (NSCLC), understanding their side effects presents a challenge for providers. Alectinib, a second-generation tyrosine kinase inhibitor, is a targeted therapy used in patients with non-small cell lung cancer found to have anaplastic lymphoma kinase (ALK) mutations. Alectinib is the focus of this case report and literature review as we seek to understand side effects providers may encounter when prescribing these therapies.

**Case presentation:**

We begin our report with the case of a 63-year-old Hispanic female with stage IIIA non-small cell lung cancer found to have the ALK genomic alteration. She was started on Alectinib, and on Day 11, she developed a severe maculopapular rash requiring hospitalization. After complete resolution, desensitization with Alectinib was attempted but unsuccessful.

**Conclusions:**

Despite the unsuccessful desensitization of this patient, it is important to report this rare side effect in order to better understand how providers can pursue management. Case reports such as this can aid providers in potentially preventing, treating, and rechallenging patients on targeted therapies in the future.

## Background

Non-small cell lung cancer (NSCLC) is one of two heterogeneously distinct subtypes and constitutes up to 76% of all lung cancer cases [1]. Unlike the other major subtype, small cell lung cancer, NSCLC has had major advancements in treatment options due to the advent of newer treatment modalities, next generation sequencing, and targeted therapy [1]. Molecular alterations in epidermal growth factor receptor (EGFR) and anaplastic lymphoma kinase (ALK) genes have allowed for management with targeted therapies.

In regards to ALK mutations, targeted therapy emerged with the first-generation tyrosine kinase inhibitor (TKIs) Crizotinib [2]. Despite the initial excitement with the use of Crizotinib in treating targetable mutations in NSCLC, it was soon after determined that this medication leads to increased rates of drug resistance [3]. Following this discovery, second-generation tyrosine kinase inhibitors Alectinib and Brigatinib made their way into the medical arena [4]. Despite the efficacy and superiority of these newer generation TKIs, there is still limited research detailing all the adverse side effects which may arise during their usage. One such adverse event that is still being understood is rash formation. Due to this, we present a case of rash formation in a patient on Alectinib and the measures that were taken in attempts for desensitization.

## Case

Herein is presented a case of a 63-year-old Hispanic female, a non-smoker with non-significant past medical history. In 2020, the patient presented to urgent care for upper respiratory symptoms. She tested positive for COVID-19, which was then treated on an outpatient basis. A month later, she returned to urgent care due to persistent dyspnea. A chest x-ray at that time revealed a lung mass, corroborated by further imaging studies, including computed tomography (CT) chest scan. Endobronchial ultrasound bronchoscopy (EBUS) was then completed which demonstrated adenocarcinoma metastatic to the right hilar and right paratracheal lymph nodes. Staging was described as IIIA non-small cell lung adenocarcinoma. Due to lymph node involvement, resection on initial diagnosis was not recommended.

At an outside facility and prior to next-generation sequencing, the patient was started on combination chemoimmunotherapy with pembrolizumab, pemetrexed, and carboplatin for 4 cycles. The reason for choosing chemoimmunotherapy over chemoradiotherapy is unknown as this was not included in patient outside records. Afterwards, single agent pembrolizumab was continued as maintenance therapy. Per outside records it appeared that the patient did not experience significant side effects on chemoimmunotherapy and the plan was to follow this treatment with surgery. Resection however, was unsuccessful and it was advised that patient have consultation at our facility. Upon arrival to our institution, results of tumor profiling revealed a PDL1 status of 99%, a low tumor mutation burden (TMB) of 3.2 Muts/mb, MSI stability, and EML-4 ALK fusion genomic variation. Due to the ALK mutation, treatment was started with a targeted agent, Alectinib at a dosage of 600 mg daily. As a result of change of care, time from her last pembrolizumab treatment to the initiation of Alectinib was 5 weeks.

**Fig. 1:**
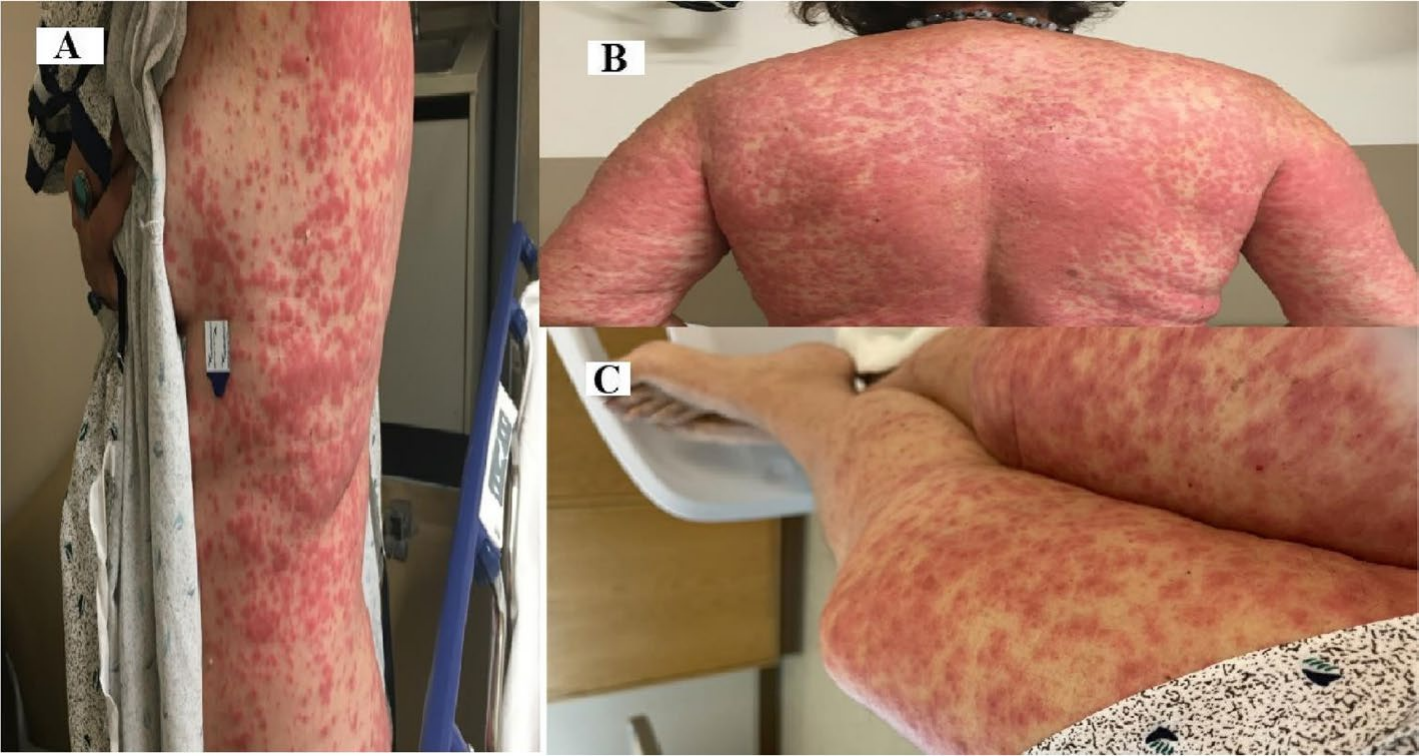
**A**, **B**, **C**: maculopapular rash

Ten days after Alectinib initiation, the patient called to report a red rash of the arms, stomach, and thighs. It was accompanied by mild fever of 99.9 Fahrenheit. She was advised to either monitor symptom progression at home or go to the emergency room. The patient chose to monitor at home. The next day the patient called to report rash progression and fevers to 101.0 Fahrenheit. She was instructed to go to the ER and hold Alectinib. Due to the severity of the rash, she was admitted to the hospital. Routine labs were normal except for mildly elevated AST at 35. The rash was described as a diffuse morbilliform eruption that was moderately pruritic (Fig. 1). She was given triamcinolone cream and Benadryl pending results of skin biopsy. Pathology results came back as basal vacuolar interface dermatitis with mild superficial perivascular and interstitial infiltrate of lymphocytes, neutrophils, and eosinophils most consistent with drug eruption. Due to this, the patient was started on methylprednisolone and discharged on prednisone taper. Within 2 weeks of initial rash presentation, the symptoms had completely resolved.

Following resolution, a desensitization protocol for Alectinib was started based on dosing found in a literature review. The agreed upon schedule with thus: 150 mg twice daily for 2 days followed by 300 mg twice daily for 5 days, then 450 mg twice daily for 8 days followed by 450 mg in the a.m. and 600 mg in the p.m. for 8 days until the standard dose of 600 mg twice daily was re-achieved (Table 1). This protocol was begun at home, with the patient given explicit instructions to notify the medical team if any symptoms arose. Within one day of restarting Alectinib, patient reported developing sore throat, erythema of the chest, and fevers of 100.1 Fahrenheit. Due to the repeated nature of symptoms, the patient was advised to discontinue Alectinib. Despite lack of rash reformation, desensitization was not continued after the development of side effects.

**Table 1:** Desensitization schedule conducted for this patient

Protocol	
**150 mg BID**	Take for 2 days
**300 mg BID**	Take for 5 days
**450 mg BID**	Take for 8 days
**450 mg QD morning 600 mg QD evening**	Take for 8 days
**600 mg BID**	Continue as standard dose

With the failure of the desensitization to Alectinib, Brigatinib was chosen due to its different chemical structure. The patient was advised to begin 90 mg daily for 1 week and to then increase to 180 mg daily at standard dosing. Patient began Brigatinib and continued for approximately 1 month with minimal side effects. However, following a trip to South America, the patient decided upon alternative management with radiation therapy. She was advised that this was not recommended in the United States due to her NSCLC staging, ALK mutation, previous immunotherapy use, and recommendations per National Comprehensive Cancer Network (NCCN) guidelines. She discontinued Brigatinib and is currently being treated outside the country. Her health status is currently unknown as she has been lost to follow-up for several months.

## Discussion

With the discovery of new treatment modalities for non-small cell lung cancer there is increased opportunity for greater progression free survival and overall survival in patients dealing with the disease [5–7]. One such modality is targeted therapy which, as suggested by its name, targets specific molecular alterations found in certain patient populations with NSCLC. One of these alterations is that of the ALK gene which causes the formation of the novel fusion oncogene EML4–ALK that is found in up to 7% of NSCLC cases [8]. Albeit in a small subset of cases, it is noted that this genetic alteration appears more frequently in patients with adenocarcinoma, female, younger age, and never to light smokers demonstrating an important mutation to search for in this cohort of patients presenting with NSCLC [9, 10].

Tyrosine kinase inhibitors have proven to be successful in treating NSCLC patients with ALK mutations. Second generation TKIs such as Alectinib and Brigatinib are quickly overtaking their predecessor Crizotinib in efficacy and superiority with increased progression free survival [11, 12]. Despite the success of these newer medications, there are still side effects from these therapies which can affect the ability of patients to continue with these treatment modalities. One of the side effects is rash presentation which, although rare, is important to identify in order to come up with the best management approach. In Alectinib usage, grade 3 level rash formation was found in only 1% of the patients as reported in the ALEX trial [11]. Within both the ALEX and J–ALEX trial, the management for the development of rash was to reduce the dosage of the medication [11, 13]. No consensus was determined in providing the best opportunity for eradication of this side effect [11, 13].

Thus, in regards to managing rash formation on Alectinib, to the best of the authors’ knowledge only four other cases have been reported of Alectinib–induced rash formation and desensitization protocols enacted to combat said reaction [14–17]. In these reports, desensitization was successful with their respective patient being able to reinitiate Alectinib treatment. Unfortunately, this was not the outcome in our case in which the patient was unable to continue with Alectinib treatment and was begun on Brigatinib. In order to avoid failure in future cases, we have gathered factors which differ in our report in comparison to the four successful cases of desensitization.

For our patient, we enacted the protocol as detailed by Seegobin et al., which differs from the other 3 cases by beginning with a higher dose of Alectinib at 150 mg twice daily [14]. The other 3 cases began at 20 mg once daily, 37.5 mg once daily, and 40 mg once daily respectively [15–17]. In our case beginning at the higher dose may have led to a greater risk of side effect recurrence. Additionally, it is important to note that in our case, a full on diagnosed rash did not occur when we reinitiated treatment. This was due to reluctance of continuing with the desensitization protocol once the initial symptoms of sore throat, erythema of the chest, and fever had returned. Other different factors were that in both reports by Shirasawa and Kimura, the patients were rechallenged with Alectinib on an inpatient basis [15, 17]. This was done in order to monitor progress and potential complications of rechallenge. In the report by Kimura et al., not only was the patient followed as an inpatient, Alectinib rechallenge was also given with 10 mg prednisolone [17]. Steroids were added in this report to help combat any other possible reaction to the re-initiation [17]. Within our case, neither of these two options were completed, as the patient was rechallenged in an outpatient setting and steroids were not administered. Combined with a higher dose on initial restart along with the decision to follow the patient on an outpatient basis may have reduced the likelihood of success in our case. Additionally, our patient had been administered 4 cycles of chemoimmunotherapy in a separate facility prior to beginning targeted therapy. It is important to realize that the amalgamation of combination chemoimmunotherapy, particularly immunotherapy, may have contributed to an adverse dermatological effect along with Alectinib [18]. As aforementioned, Brigatinib was then chosen due to its differing chemical structure. To corroborate likely success of switch there are at least two other cases of successful changes from Alectinib to Brigatinib due to serious dermatological reactions [19, 20]. Unfortunately, understanding that relation will never be accomplished as a result of loss of contact with the patient following her move out of the country.

Understanding the limitations of our case; and that the exact reasons for failure of desensitization cannot be known; it is important that we discuss potential causes in order to avoid repeated cases of failure. It is important for each physician to tailor regimens to the needs of the patient. With continued research on the subject the more likely it is to determine what factors work best for ensuring success in order that the patient may continue Alectinib treatment. With this in mind, we suggest the physicians consider rechallenging with a lower dose and titrating upwards to full dosage on an inpatient basis. Additionally, the use of steroids proved beneficial in the report by Kimura et al., who followed the protocol as seen in the successful re-initiation of Sorafenib in a case of rash formation in a patient with metastatic renal cell carcinoma [17, 21]. This is not the first time that the addition of steroids to rechallenge therapy has proved successful; as it has also been documented in treatment of other adverse grade events including Osimertinib-induced interstitial lung disease [22]. Thus, we suggest the consideration of steroid usage on a case–by–case basis for patients presenting for rechallenge.

## Conclusion

As the leading cause of cancer worldwide, lung cancer, including its most common subtype non-small cell, is a formidable disease that is very difficult to treat. Until recently, not much had changed in terms of management options for those diagnosed. Within the last few decades however, came the advent of newer therapies that brought the excitement of increased survival and a new hope for patients. With these new treatment modalities came the complication of recognizing more challenging side effects to overcome. Because of the positive findings of these newer treatments in prolonging survival and quality of life it is very important that physicians be able to better understand the side effects and how to properly manage them when they are found. Thus, the more cases reported on such side effects lends to a greater degree of ways to manage them**.**

## Data Availability

Not applicable.

## Abbreviations

NSCLC: Non-small cell lung cancers
ALK: Anaplastic lymphoma kinase
EGFR: Epidermal growth factor receptor
TKIs: Tyrosine kinase inhibitor
CT: Computed tomography
EBUS: Endobronchial ultrasound bronchoscopy
TMB: Tumor mutation burden
NCCN: National Comprehensive Cancer Center network
